# Haste Makes Waste but Condition Matters: Molt Rate–Feather Quality Trade-Off in a Sedentary Songbird

**DOI:** 10.1371/journal.pone.0040651

**Published:** 2012-07-12

**Authors:** Csongor I. Vágási, Péter L. Pap, Orsolya Vincze, Zoltán Benkő, Attila Marton, Zoltán Barta

**Affiliations:** 1 Department of Evolutionary Zoology, University of Debrecen, Debrecen, Hungary; 2 Evolutionary Ecology Group, Hungarian Department of Biology and Ecology, Babeş-Bolyai University, Cluj Napoca, Romania; 3 MTA-DE “Lendület” Behavioural Ecology Research Group, University of Debrecen, Debrecen, Hungary; University of Western Ontario, Canada

## Abstract

**Background:**

The trade-off between current and residual reproductive values is central to life history theory, although the possible mechanisms underlying this trade-off are largely unknown. The ‘molt constraint’ hypothesis suggests that molt and plumage functionality are compromised by the preceding breeding event, yet this candidate mechanism remains insufficiently explored.

**Methodology/Principal Findings:**

The seasonal change in photoperiod was manipulated to accelerate the molt rate. This treatment simulates the case of naturally late-breeding birds. House sparrows *Passer domesticus* experiencing accelerated molt developed shorter flight feathers with more fault bars and body feathers with supposedly lower insulation capacity (i.e. shorter, smaller, with a higher barbule density and fewer plumulaceous barbs). However, the wing, tail and primary feather lengths were shorter in fast-molting birds if they had an inferior body condition, which has been largely overlooked in previous studies. The rachis width of flight feathers was not affected by the treatment, but it was still condition-dependent.

**Conclusions/Significance:**

This study shows that sedentary birds might face evolutionary costs because of the molt rate–feather quality conflict. This is the first study to experimentally demonstrate that (1) molt rate affects several aspects of body feathers as well as flight feathers and (2) the costly effects of rapid molt are condition-specific. We conclude that molt rate and its association with feather quality might be a major mediator of life history trade-offs. Our findings also suggest a novel advantage of early breeding, i.e. the facilitation of slower molt and the condition-dependent regulation of feather growth.

## Introduction

The trade-off between current reproductive effort and future (residual) reproductive value is of outstanding concern in life history theory [1], [2]. However, the mechanisms that potentially mediate such long-term effects remain an evolutionary conundrum. It was recently proposed that molt (regular replacement of worn and torn feathers) and the quality of the feathers produced in particular may couple events that precede and follow the molting period via carry-over effects (‘molt constraint’ hypothesis, MCH; [3], [4]). The MCH suggests that the long-term costs of breeding are expressed via a compromised molt, which can lead to plumage malfunctions and hence curtailed future fitness.

The MCH integrates several lines of evidence. First, it assumes that breeding and molt are scheduled sequentially during the annual cycle due to the adaptive avoidance of overlapping two costly activities [5], [6], which holds for most temperate zone bird species [7], [8]. Thus, prolonged breeding due to its late onset or higher investment evokes delayed molt [9]–[11]. Second, molting is a costly process [12]–[14], and molting birds may encounter elevated energetic constraints due to lower resource availability if the initiation of molt is postponed [3]. Consequently, any delay might manifest in the production of functionally inferior feathers. This may be exacerbated because the approaching winter shortens the time available for plumage exchange, which forces birds to accelerate their rate of molt [4], [8]. However, this seems a best-of-a-bad-job strategy because the rate of molt is traded off against the quality of flight feathers [4], [15]–[19] and presumably the structure of body feathers as well [3], [20]. Third, poor-quality feathers might not serve their main functions adequately, e.g. insulation, flight and communication. These losses of function are obviously precipitated in fitness reduction by abating future performance [3], [7]. Compromised molt (1) impinge fundamental adverse effects on flight [21]–[23] and thermoregulation [3], [20] due to lower-quality feathers, (2) diminishes reproductive success because of breakage or altered pigmentation and/or microstructure of feathers involved in visual displays ([24] and refs therein, [25]), or (3) might have multiple costs through starvation–predation and thermoregulation–predation trade-offs [26], [27]. In conclusion, this cause–effect cascade implies that the molting process might link past (or current) and future life history events.

The MCH has mainly been studied by the correlative approach on the flight feathers of migratory species ([16]–[18]; see [15] for an experiment). Exceptions include comparisons of migratory and sedentary blackcap *Sylvia atricapilla* populations (e.g. [17], [28]) and two experiments with European starlings *Sturnus vulgaris* in East England [4], [19]. Although starlings are sedentary or partial migrants in Britain, their annual routine resembles that of summer-molting migrants with a short breeding period (usually only one clutch in East England) and they shed their plumage early (May–August) [29]. Temperate zone migrants commonly employ the summer-molting strategy [30] that spans a shorter period and results in the production of lighter feathers compared with sedentary birds [17], [18]. In contrast, sedentary birds partition their annual cycle into fewer costly activities (lacking migration), which allows more time for plumage renewal [8], [31]. An open question is whether the inter-individual variation in molt rate and duration in a less time-constrained sedentary population is sufficiently large to generate variation in feather quality. Furthermore, only one correlative study of sedentary great tits *Parus major* dealt with molt constraints on body feather quality [20]. To the best of our knowledge, no experimental studies have explored whether the accelerated molt rate can represent one of the proximal underpinnings of altered body feather structure, which is one of the cornerstones of the MCH [3], [20]. Altogether, this topic clearly deserves further investigation. The house sparrow *Passer domesticus* is a ‘typical’ sedentary bird; it is multi-brooded (up to four clutches in Central and Eastern Europe; [29]), consequently its reproductive period is much longer and hence it molts later (August–early November in our wild-living population; Pap PL, Vágási CI, Barta Z unpublished data) than similar-sized migratory species. This makes it an ideal candidate for testing this hypothesis.

Body condition is a phenotypic trait, which refers to size-adjusted, non-skeletal mass and it was defined by Peig and Green [32] as “the energy capital accumulated in the body … assume[d] to be an indicator of an animal’s health and quality.” Variation in body condition has a substantial heritable component in a wide range of taxa ([33] and refs therein) including the house sparrow [34]. Thus, body condition might have broad evolutionary implications because it shapes a plethora of life history and fitness-related traits [2], [33], [35]–[37]. Molt is not an exception; a trade-off has been documented between body mass (i.e. mass unadjusted to size) and molt rate, and body mass also mediates the molt–immunity trade-off [14]. Further, body mass and body condition are also relevant to the MCH because body mass can predict molt initiation [5], while body condition can predict speed of molt [38]. However, body mass does not correlate with feather growth rate [39]. Each metabolically costly activity has recourse to finite stores, so birds in a better condition can afford higher investment due to their higher amount of resources available for supporting demanding activities and/or their more efficient replenishment of stores after allocation (higher turnover rate). Embedded in the molt constraint scenario, fitness accrual can be mediated by body condition if birds with greater energy capital can better fuel the demands of molting to grow better-quality feathers, even if molt is compromised (i.e. show higher developmental homeostasis). Yet this aspect has only been addressed indirectly [17] or investigations have been limited to feather growth rate [39], [40] and molt–immunity trade-off [14].

Our previous study showed that house sparrows subjected to a photoperiod regime that simulated a late molting season experienced an accelerated molt of flight feathers, wing coverts and body feathers of the melanin-based throat patch (badge of status) [24]. These indicated that the overall plumage molting program was affected by the treatment. Fast-molting birds renewed their plumage approximately two weeks earlier than controls, although molt initiation was similar in the two groups, and grew a smaller badge and a less bright wing-bar. The current study continued this experimental investigation of the molt pattern in the same birds, but the aim was to determine whether life history trade-offs might be mediated by molt rate. The following predictions were tested. First, the speed of molt should have no effect on feather attributes if the longer molting period of sedentary birds permits cost-spreading. Second, Broggi et al. [20] found that northern great tits, which face time constraints grew shorter, more densely structured body feathers with fewer plumulaceous barbs. It is suggested that such feather architecture may provide less efficient heat conservation ([3], [20] and refs therein). If the MCH of Nilsson and Svensson [3] stands, the body feather structure of fast-molting experimental sparrows should resemble that of northern great tits. This is a missing piece of the MCH. Third, an interaction between treatment and body condition is predicted if good-quality birds can cope better with a faster molt rate.

## Materials and Methods

### Ethics Statement

Birds were handled in strict accordance with animal care, wellbeing and ethical prescriptions [41]. The protocol for bird care and experimentation adhered to the current Romanian laws and was approved by the Romanian Academy of Sciences (permit number: 2257).

### General Procedures

We caught 50 non-molting adult male house sparrows at a cattle farm near Cluj Napoca (46°46′N, 23°33′E, Transylvania, Romania) on 25 July 2008. Birds were transferred to the Campus of Babeş-Bolyai University, Cluj Napoca and randomly assigned in equal numbers into two indoor aviaries (each 4 m L×3.5 m W×4 m H). Birds allocated to the aviaries did not differ in any measured aspect (one-way ANOVA, pre-molt wing length: *F*_1,47_ = 0.09, *P* = 0.76; pre-molt tail length: *F*_1,43_ = 0.22, *P* = 0.64; body mass: *F*_1,44_ = 1.47, *P* = 0.23; tarsus length: *F*_1,47_ = 0.14, *P* = 0.71). During confinement, birds had *ad libitum* access to protein-rich food (seed mixture supplemented with grated boiled eggs or mealworms on odd days; [13], [24]), sand and daily exchanged drinking water. Aviaries contained bushes, perches and nest boxes. Coccidian parasites that emerge spontaneously in captive populations were purged by an anticoccidial drug (toltrazuril) administered in the drinking water [24]. Except one sparrow from the control group that died for unknown reasons, the rest of the birds were released back to the population of origin in good body condition after the termination of the experiment (on 15 November 2008).

Aggressive behavior can determine resource acquisition and has physiological bases (e.g. steroid hormones) that are recognized to influence keratin synthesis. Still, group housing was issued because free-ranging house sparrows are gregarious in the molting period [29]. To preclude the possible confounding effects caused by male–male interactions, we recorded within flock agonistic behavior of individually marked sparrows from a hide, calculated fighting success (% fights won out of total) and included this in all subsequent statistical analyses (see [24] for details).

### Photoperiod Treatment

**Figure 1:**
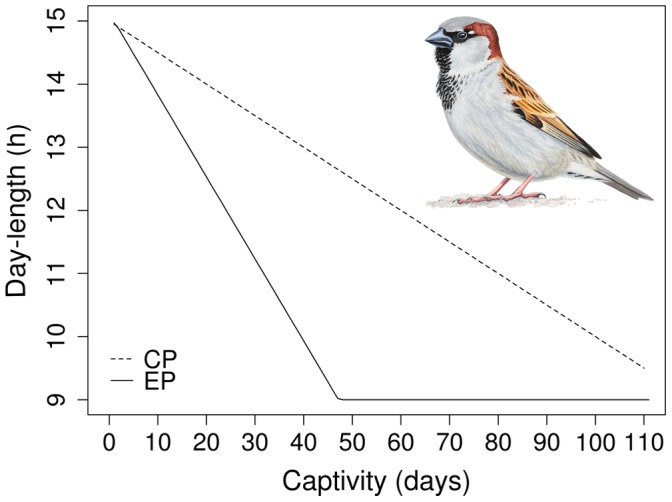
Photoperiod regime at which male house sparrows undertook their molt. Photoperiod treatment groups: ‘CP’ control (broken line), ‘EP’ experimental (continuous line). 28 July 2008 =  day 1, 25 November 2008 =  day 110. Male house sparrow drawing credit: Márton Zsoldos.

Birds were subjected to a photoperiod treatment consisting of two different lighting regimes. At capture, the local natural photoperiod was 15L:9D (light:dark). The lighting regime of control group birds was a steadily decreasing photoperiod that conformed the natural photoperiod characteristic to study latitude (control photoperiod, hereafter ‘CP’ group). This meant a decrease in day-length by in average 3 min per day. Birds of the experimental group experienced an accelerated seasonal decrease in photoperiod (experimental photoperiod, hereafter ‘EP’ group; 8 min decrease in day-length per day) until reaching 9L:15D photoperiod at the 46th day of experimentation and then held it constantly at this level (Fig. 1). This setup simulates photoperiod conditions met by early-molting (CP) and late-molting (EP) sparrows. We concomitantly followed the molting pattern of the field population and found this to be similar to that of CP birds (personal observation), indicating that captivity did not alter their normal molting pattern.

### Measurements

Wing and tail length (±0.5 mm) were measured with a ruler both pre- and post-molt. At capture and on every 10th day of the 110-day-long experimentation, body mass (±0.1 g) was measured with a Pesola spring balance and molt status was scored (see below). Hereafter, ‘mass’ is shorthand for “mean body mass averaged over the 11 measurement sessions”. Adult house sparrows perform one post-breeding molt per year, thus feathers developed in a given year are retained until the end of breeding in the next year. Only the molting status of primaries was scored because it gives a good general picture about the complete post-breeding molt [13]. The molt status of each primary on both wings was scored on a 0–5 scale according to Ginn and Melville [8] from which the molting index was derived as the sum of 9 primaries’ scores per wing (range 0–45). Note that the outermost primary is rudimentary in sparrows. Molt of flight feathers starts with the innermost primary. Molt was considered to be started on the day when the molt index was first >0. The reaching of a molt score of 45 marks the end of molt. The duration of molt is the number of days elapsed between molt onset and molt termination.

Once molt was completed, we plucked the 5th and 7th primaries (henceforth P5 and P7) of both wings. We use descendant primary numbering, i.e. the 1st primary, P1, is the innermost. The rationale behind choosing these feathers stems from the followings: (1) P5s were grown when groups started to diverge in molt speed, whereas P7s when the difference in molt speed was the largest between groups (see Fig. 1 in [24]), and (2) P5s grow at the highest rate, whereas P7s are the longest primaries thus necessitating the most keratin, though developing at a slower pace [13]. We further removed 4–5 contour body feathers from the mantle region. All feathers were stored dry in zip-lock plastic bags. The quality of flight feathers was quantified through 4 parameters (mass, length, the width of the rachis and the number of fault bars; [4], [13], [21], [42], [43]), whereas that of body feathers through 5 parameters (barbule density, total number of barbs, proportion of plumulaceous barbs, length and area; [20]). The rachis is the solid upper part of the feather shaft from which barbs are branching. Barbs have further branches, called barbules that connect the adjacent barbs to form together the feather vane of pennaceous body and flight contour feathers. The measures of left and right wing primaries and that of 2–3 randomly chosen body feathers were averaged to increase measurement accuracy. To reduce measurement error, each trait was assessed by one author (CIV, Z. Benkő or AM) without knowledge on group identity of samples.

Dry mass of primaries was measured with a SCALTEC (SBA 32) balance (±0.1 mg). Maximum length of primaries (from the proximal end of calamus to tip) was measured with a ruler (±0.5 mm) by stretching the feather straight. Rachis width of primaries was measured at the base of the vane with a digital caliper (±0.01 mm). The measurement of primary mass, length and rachis width proved to be highly repeatable within individuals (*R*>0.8) in a previous study with the same methodology [13]. Fault bars are abnormalities that appear in form of translucent bands quasi perpendicular to the rachis where feathers are prone to break [21]. These were counted on all 4 plucked primaries and summed up. Barbule density of body feathers was measured by taking digital photographs under a stereomicroscope (Olympus SZ61, Tokyo, Japan; camera: Cool SNAP-Pro cf) at 50× magnification. Feathers were placed in a frame fixed to microscope platform aspiring to expose the same feather region. Feathers were sandwiched between two microscope slides to flatten the naturally curved feathers rendering possible planar measurements. Barbules were counted at the three longest barbs along 1.5 mm length of barb starting from the rachis. The standardized 1.5 mm lengths, calibrated from a stage micrometer scale, were traced in ImageJ [44]. When counting the number of barbs we progressed along the shaft at 30× magnification and made a distinction between pennaceous and plumulaceous barbs according to Broggi et al. [20]. Pennaceous barbs are compact, closer to each other, have shorter barbules and often form vanes. Plumulaceous barbs are fluffy, with long barbules and do not create vanes, hence trap air for insulation. We recorded the total number of barbs and that of pennaceous ones and calculated the proportion of plumulaceous barbs (%) as (total barbs – pennaceous barbs)/total barbs. For length and area, mantle feathers were photographed (Nikon D80 on tripod) on a grey-card with metric template and sandwiched between two microscope slides. Feather length (±0.01 mm) was measured by tracing a line along the shaft (calamus included), whereas area (±0.01 cm^2^) by encircling the feather in ImageJ.

### Statistical Procedures

All statistical analyses were performed in the R statistical environment version 2.14.1 [45]. We built linear models (LMs) separately for each response variable except the total number of fault bars, which was analyzed by generalized linear model (GLM) with Poisson error distribution. Treatment was entered as factor and the correlated trait, if any, of focal variables as covariate to deal with the few inter-correlations between response variables. In addition, scaled mass index (SMI; [32]) and square root-transformed fighting success were also entered as covariates. SMI 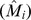 is a size-corrected body condition index calculated as
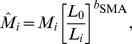
where *M_i_* and *L_i_* are the mass and tarsus length of *i*th individual, respectively, *L*_0_ is the mean tarsus length of the sample and exponent *b*_SMA_ is the slope of log-log model II standardized major axis (SMA) regression of mass on tarsus length (‘lmodel2’ package for R; [46]). To test whether treatment has a condition-dependent effect, the treatment × SMI interaction term was also entered. Because the widely used condition proxy computed as residuals from mass on skeletal length ordinary least squares regression violates certain statistical assumptions [37], we calculated SMI (g) as this more accurately indicates non-skeletal mass. SMI was scaled to mean = 0 in each model by subtracting the sample’s mean SMI from each individual’s value in order to gain meaningful parameter estimates. Entering both SMI and fighting success as continuous predictors is reasonable because they are unrelated (Pearson’s product-moment correlation, *r* = 0.01, *n* = 49, *P* = 0.98). We first built saturated models that were simplified to minimum adequate models (MAMs) by backward stepwise procedure dropping the predictor with lowest improvement of model fit. For this selection we chose the *P*<0.1 criteria to retain confounding variables with weaker influence. MAMs were not allowed to differ from the saturated model in explanatory power. The treatment main effect was always kept in the model, while SMI only when it was significant itself or non-significant itself but significantly interacted with treatment. We adopt stepwise elimination as it performs similarly as information theory approach [47]. The requirements of MAMs were checked by plot diagnosis. Each model suited the requirements of linearity, residual’s variance and distribution, and no outliers (i.e. standardized residuals > |3|) were detected.

Only the MAMs are presented. Tests are two-tailed and type I error probabilities were set to 0.05. Mean ± SE of raw data and model estimate *b* ± SE for covariates are reported throughout. Sample sizes are *n*_CP_ = 24 and *n*_EP_ = 25 for each variable except for post-molt tail length for which *n*_CP_ = 21 and *n*_EP_ = 24. We were unable to measure post-molt tail length for some birds because of broken feathers. Repeatability calculations were performed as per Nakagawa and Schielzeth [48] using the ‘rptR’ package for R and restricted maximum likelihood method.

## Results

Measured body feather parameters were highly repeatable (*n* = 7 randomly remeasured individuals; barbule density: *R* = 0.87, 95% CI = 0.67–0.91, *P* = 0.0002; total number of barbs: *R* = 0.74, 95% CI = 0.26–0.89, *P* = 0.007; proportion of plumulaceous barbs: *R* = 0.73, 95% CI = 0.26–0.92, *P* = 0.006; length: *R* = 0.92, 95% CI = 0.70–0.99, *P* = 0.0002; area: *R* = 0.80, 95% CI = 0.22–0.91, *P* = 0.0002). Further, the within-individual values of the 2–3 feathers were significantly correlated (Pearson’s product-moment correlation, all *n* = 49; barbule density: *r* = 0.36, *P*<0.01; total number of barbs: *r* = 0.57, *P*<0.0001; proportion of plumulaceous barbs: *r* = 0.45, *P*<0.001; length: *r* = 0.70, *P*<0.0001; area: *r* = 0.63, *P*<0.0001).

Tarsus length (mm) in our study population was 19.38±0.09 and the slope of log-log mass on tarsus length SMA regression was 1.17. Thus, SMI (g) was computed as mass*_i_* × (19.38/tarsus length*_i_*)^1.17^ and averaged 29.00±0.16. Groups did not differ in SMI (LM, *F*_1,47_ = 0.10, *P* = 0.76), indicating that photoperiod manipulation had influenced molt speed [24] without deteriorating the mean body condition of birds.

### Post-molt Wing and Tail Length, and Fault Bars

**Figure 2:**
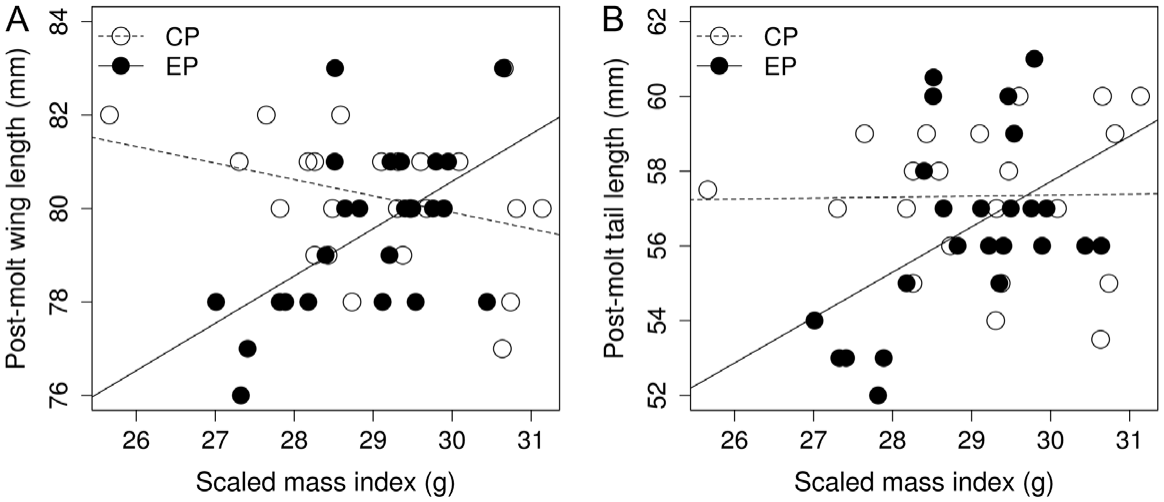
Photoperiod treatment effects on post-molt morphology. Panels show the treatment by condition (scaled mass index) interaction on post-molt length (mm) of (A) wing and (B) tail. Photoperiod treatment groups: ‘CP’ control (open circles, broken line), ‘EP’ experimental (filled circles, continuous line).

Treatment had no effect on post-molt length (mm) of wing (CP: 80.25±0.29; EP: 79.52±0.35) and tail (CP: 57.33±0.43; EP: 56.44±0.50), but interacted significantly with SMI (Table 1) indicating that CP bird’s wing and tail length is unrelated to condition (wing: *b* = –0.40±0.26, *t* = –1.54, *P* = 0.13; tail: *b* = 0.03±0.40, *t* = 0.08, *P* = 0.94), whereas in the EP group only birds in good condition grew long wings and tail (wing: *b* = 1.14±0.43, *t* = 3.61, *P* = 0.0008; tail: *b* = 1.31±0.65, *t* = 2.08, *P* = 0.04) (Fig. 2). Fighting success was dropped from all models.

**Table 1:** Minimum adequate models (MAMs) on the significant or marginally significant predictors of several morphological and flight feather traits.

*Response*/predictor	MS	*F*	df	*P*
*Post-molt wing length*				
SMI a	1.60	0.79	1,45	0.38
TREAT	6.25	3.09	1,45	0.09
SMI × TREAT	26.36	13.03	1,45	0.0008
MAM: error MS = 2.02, *F*_3,45_ = 5.64, *P* = 0.002, *R* ^2^ = 0.27				
*Post-molt tail length*				
SMI	14.91	3.30	1,41	0.08
TREAT	7.42	1.64	1,41	0.21
SMI × TREAT	19.46	4.31	1,41	0.04
MAM: error MS = 4.51, *F*_3,41_ = 3.09, *P* = 0.04, *R* ^2^ = 0.18				
*Length of P5*				
SMI	1.64	0.70	1,45	0.41
TREAT	6.63	2.82	1,45	0.10
SMI × TREAT	16.18	6.88	1,45	0.01
MAM: error MS = 2.35, *F*_3,45_ = 3.46, *P* = 0.02, *R* ^2^ = 0.19				
*Length of P7*				
SMI	1.25	0.76	1,45	0.39
TREAT	11.70	7.16	1,45	0.01
SMI × TREAT	19.59	11.99	1,45	0.001
MAM: error MS = 1.63, *F*_3,45_ = 6.64, *P* = 0.0008, *R* ^2^ = 0.31				
*Rachis width of P5*				
SMI	0.01	4.65	1,46	0.04
TREAT	0.01	0.06	1,46	0.81
MAM: error MS = 0.01, *F*_2,46_ = 2.35, *P* = 0.11, *R* ^2^ = 0.09				
*Rachis width of P7*				
TREAT	0.01	2.06	1,47	0.16
MAM: error MS = 0.01, *R* ^2^ = 0.04				

a Photoperiod treatment (‘TREAT’) effects are shown even if not significant, whereas condition (‘SMI’, scaled mass index (g)) was retained only when significant but also when interacted with TREAT.

Total number of fault bars was significantly higher in the EP (1.52±0.24, median = 1.0, range = 0–5) than in the CP group (0.38±0.12, median = 0.0, range = 0–2; GLM, *χ* ^2^_1_ = 18.08, *P*<0.0001). None of the other predictors were retained in the MAM.

### Flight Feathers

**Figure 3:**
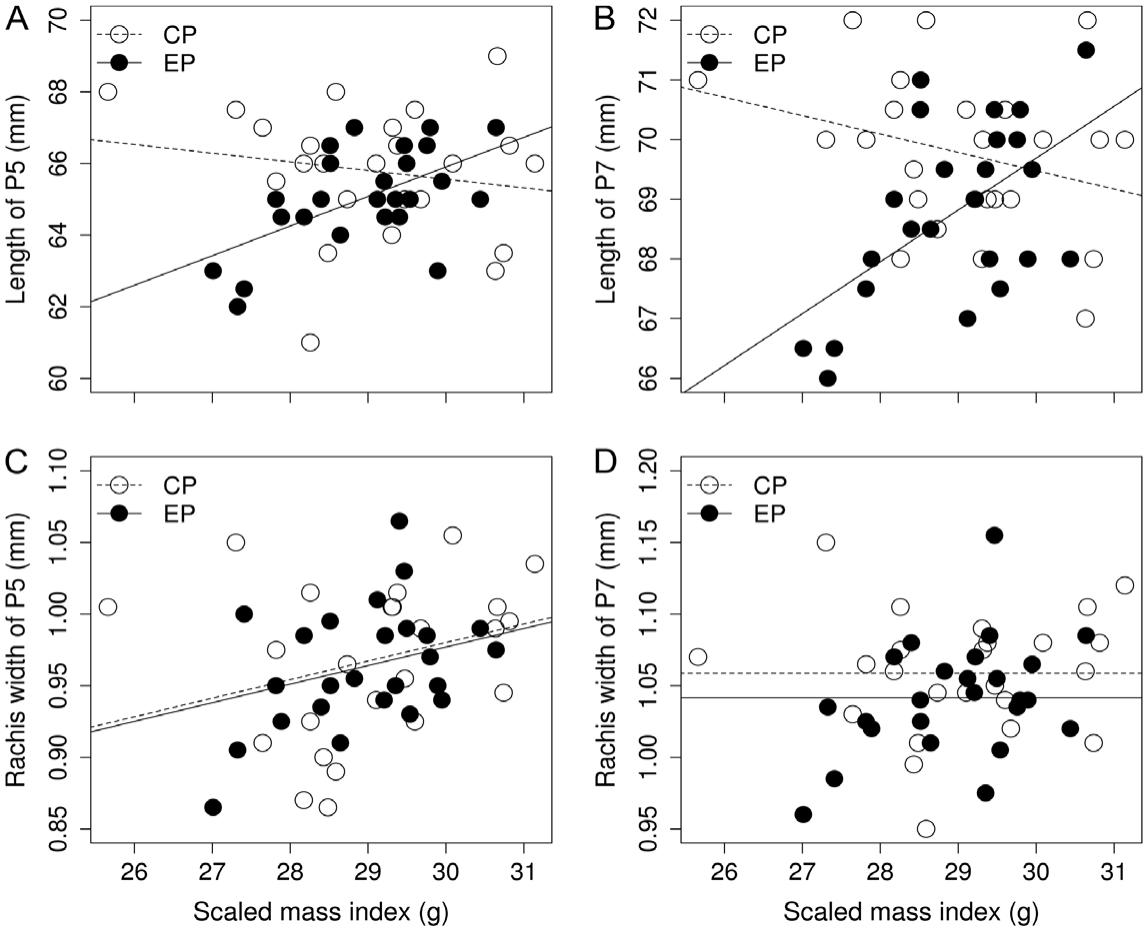
Photoperiod treatment effects on flight feather traits. Left side figures depict the effects of the treatment by condition (scaled mass index) interaction on length (A) and rachis width (C) of P5, whereas the right side figures (B, D) show the same attributes of P7. Photoperiod treatment groups: ‘CP’ control (open circles, broken line), ‘EP’ experimental (filled circles, continuous line).

Because feather mass and length were strongly positively correlated (P5: *b* = 0.39±0.06, *t* = 6.70, *P*<0.0001; P7: *b* = 0.36±0.08, *t* = 4.65, *P*<0.0001) and treatment had qualitatively similar results on these two parameters, we show only results concerning feather length. Groups did not differ in the length (mm) of P5 (CP: 65.79±0.37; EP: 65.04±0.28) and P7 (CP: 69.77±0.27; EP: 68.78±0.30), but treatment interacted with SMI (Table 1). This indicates that feather length was unrelated to condition in the CP group (P5: *b* = –0.27±0.28, *t* = –0.98, *P* = 0.33; P7: *b* = –0.35±0.23, *t* = –1.50, *P* = 0.14), whereas a positive association was found in the EP group (P5: *b* = 0.94±0.46, *t* = 2.62, *P* = 0.01; P7: *b* = 0.98±0.46, *t* = 3.46, *P* = 0.001) (Fig. 3). The rachis width (mm) of P5 was not affected by treatment (CP: 0.97±0.01; EP: 0.96±0.01), but was positively related to body condition (*b* = 0.02±0.01; Table 1, Fig. 3C). The rachis width of P7 was unaffected either by treatment (CP: 1.06±0.01; EP: 1.04±0.01) or by other predictors (Table 1, Fig. 3D). Fighting success was never retained in the MAMs.

### Body Feathers

**Figure 4:**
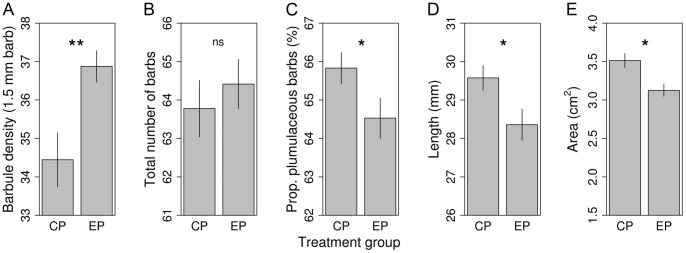
Photoperiod treatment effects on body feather traits. Panels show the effect of treatment on the density of barbules (A), total number of barbs (B), proportion of plumulaceous barbs (C), length (D) and area (E) of mantle feathers (mean ± SE). Photoperiod treatment groups: ‘CP’ control, ‘EP’ experimental. ‘ns’ non-significant, ‘*’ *P*≤0.05, ‘**’ *P*<0.01.

Structure of mantle feathers was determined chiefly by treatment (Table 2). Fast-molting EP birds produced feathers with higher barbule density per 1.5 mm barb length (CP: 34.44±0.71; EP: 36.87±0.41; Fig. 4A). The total number of barbs was marginally positively related to mantle feather length (*b* = 0.50±0.27; Table 2), hence we controlled for feather length in the model. Groups were similar in total number of barbs (CP: 63.78±0.73; EP: 64.41±0.64; Fig. 4B). The results remained unchanged when the control for the marginal effect of feather length was omitted (not shown). Fast-molting EP birds had lower proportion (%) of plumulaceous barbs (CP: 65.83±0.40; EP: 64.53±0.52; Fig. 4C). Those sparrows developed shorter mantle feathers (mm) that were in the EP group (CP: 29.58±0.32; EP: 28.36±0.41; Fig. 4D) and were in lower condition (*b* = 0.51±0.26; Table 2). The area (cm^2^) of mantle feathers was strongly positively predicted by its length (*b* = 0.16±0.02, *t* = 6.49, *P*<0.0001). Treatment influenced mantle feather area by EP birds having smaller feathers (CP: 3.51±0.09; EP: 3.12±0.08; Fig. 4E) even after controlling for length (Table 2). Thus, EP birds had smaller feathers in terms of both length and width. None of the body feather parameters were related to fighting success.

**Table 2:** Minimum adequate models (MAMs) on the significant or marginally significant predictors of several body (mantle) feather traits.

*Response*/predictor	MS	*F*	df	*P*
*Barbule density*				
TREAT a	72.25	9.05	1,47	0.004
MAM: error MS = 7.99, *R* ^2^ = 0.16				
*Total number of barbs*				
Length of mantle feather	27.15	2.46	1,46	0.07
TREAT	16.96	1.54	1,46	0.22
MAM: error MS = 11.02, *F*_2,46_ = 2.00, *P* = 0.15, *R* ^2^ = 0.08				
*Proportion of plumulaceous barbs*				
TREAT	20.77	3.84	1,47	0.05
MAM: error MS = 5.41, *R* ^2^ = 0.08				
*Length*				
SMI	13.65	4.33	1,46	0.04
TREAT	16.75	5.31	1,46	0.03
MAM: error MS = 3.16, *F*_2,46_ = 4.82, *P* = 0.01, *R* ^2^ = 0.17				
*Area corrected to length*				
Length of mantle feather	5.25	57.73	1,46	<0.0001
TREAT	0.43	4.77	1,46	0.03
MAM: error MS: 0.09, *F*_2,46_ = 31.25, *P*<0.0001, *R* ^2^ = 0.58				

a Photoperiod treatment (‘TREAT’) effects are shown even if not significant, whereas condition (‘SMI’, scaled mass index (g)) was retained only when significant but also when interacted with TREAT.

## Discussion

We showed that a simulated time-constraint was effective in triggering different molting patterns [24] within the bounds found in free-living birds ([29] and personal observation). The more rapid molt of experimental birds was detrimental for flight and body feather quality, which might ultimately have fitness costs (see Introduction). Moreover, the fitness costs of lower quality flight and body feathers may be exacerbated over time because the non-living tissue of poor-quality feathers deteriorates to a greater extent between molts [4], [16], [43]. Note that these results were obtained using a relatively mild photoperiod treatment (confer with [4]) and a sedentary model organism. We conclude that the sedentary lifestyle does not equate to release from the molt rate–feather quality trade-off. The experimental birds harbored more fault bars (also known as stress bars), which might indicate that accelerated molting was stressful [49]. This suggests that corticosterone (‘stress hormone’) may be a mediator of the molt rate–feather quality trade-off, as corticosterone is known to affect a wide range of feather attributes [50]. Importantly, these results were not confounded by aggression between birds housed in flocks in accordance with our previous work [24] where groups did not differ in the frequency of fighting, and fighting success did not affect plumage ornament expression.

Birds that underwent an accelerated molt developed shorter flight feathers. These results agree with and complement previous studies on the molt rate–feather quality trade-off in migratory or partially migratory species [4], [15]–[19]. An experimentally accelerated molt resulted in shorter and lighter primaries in starlings, and shorter primaries and wings in lesser whitethroats *Sylvia curruca* [4], [15], [19]. A faster molt resulted in less durable primaries and a greater loss of wing length between molts in grey plovers *Pluvialis squatarola* [16], lighter feathers in summer-molting passerines [17], [18], and thinner, but surprisingly more rigid (stiffer), rachis in blackcaps [28]. The molt rate is not the only driver of feather quality because other challenges during molt alter the same feather traits in a similar direction, e.g. infestation [42]. Given that investment in molt and immune function interfere [14], suppressed immunity and consequent higher susceptibility to parasites may indirectly connect molt with feather quality. The rachis width was not influenced by molt rate *per se* or its interaction with body condition. The second moment of the cross-sectional area of the rachis is related to its material rigidity [51], so we infer that this was probably less likely to be affected by the molt rate of a sedentary species as opposed to a more time-constrained migratory species ([4]; but see [28] for how selection on migratory species that rely on assiduous long-distance flights might have led to the evolution of high flexural stiffness).

This study is the first, as far as we are aware, to test the effects of molt speed on body feather structure using an experimental approach that enables the inference of causality. Rapid molting resulted in shorter and smaller feathers with a higher barbule density and a lower proportion of plumulaceous barbs [20]. This feather architecture is proposed to have a lower capacity for heat conservation because it captures less air due to inferior coverage by small feathers and its less fluffy structure (fewer plumulaceous barbs and a higher barbule density; [20] and refs therein). Thus, this study provides experimental support for the underlying mechanism proposed by Nilsson and Svensson [3] and the supposition of Dawson et al. [4] and Broggi et al. [20] that the MCH might also apply to body feathers. Our results entirely corroborate those found by Broggi et al. [20] and highlight that merely an accelerated molt rate could be sufficient to produce the inter-population differences in contour body feather structure found in great tits. Thus, great tits living on the northern margins of their European distribution might develop feathers with an impaired insulation capacity because of a time-constrained molt. As a consequence, fast-molting birds might expend more energy during thermal challenges [3], [52]. These molt rate-mediated costs could influence population processes and be responsible for the sink population attributes (e.g. lower survival rate) in the northern margins of the distribution range of the great tit [20].

An interesting aspect we found is that body condition was positively associated with feather traits either irrespective of the treatment (primaries’ rachis width and mantle feather length) or only in the experimental group (wing, tail and primary lengths). Therefore, feather traits could be honest indicators of the bearer’s condition (Figs. 2,3) in concert with Moreno-Rueda’s [14] proposal. Birds can achieve functionally adequate plumage by starting to molt early in the season, but only birds with a good body condition can buffer against a compromised molt (interactions on Figs. 2,3) if they “miss the boat” of early molt. Nestling growth and body condition have been under scientific siege in avian evolutionary ecology for a long while. Early-life growth costs were addressed frequently by means of brood size manipulation experiments (e.g. [53]). A general pattern emerged where growth-related costs were masked under good environmental condition (brood size reduction), whereas they were ubiquitous in the opposite situation (brood size enlargement). To the best of our knowledge, this is the first report of the body condition-dependent costs of somatic growth during adulthood using a manipulative tool. Under good environmental condition (relaxed molt), sparrows were capable of growing good-quality feathers independent of their body condition. However, only birds with a good body condition were capable of doing so in a challenging situation (rapid molt). In other words, good condition birds had narrower reaction norms (Figs. 2,3); they could withstand challenging environmental circumstances better and by virtue of their higher quality they endure the increased demands of molt (i.e. show higher developmental homeostasis). These conclusions agree with de la Hera et al.’s [17] graphical model, as well as the molt–mass trade-off and mass-mediated molt–immunity trade-off reported by Moreno-Rueda [14]. We argue that the link between feather quality and body condition persists outside the molting period owing to a positive feedback loop across years. That is, the growth of low-quality feathers in a given year is followed by lower condition and breeding performance in the next year, which compromises the molt once again as a consequence (this is referred to as the ‘running wheel’ effect because of the predictable cycle of states in sequential life stages). At least two lines of empirical evidence support this reasoning. First, feather quality depended on condition-indicator traits throughout the entire annual cycle of great tits [43]. Second, feather traits were repeatable over years within individual blackcaps [54]. Low-quality birds in particular are most probably trapped in this ‘running-wheel’ because they are often late breeders and thus have deferred molt onset [55], [56]. Understanding how individuals carry over effects from one season to the next has crucial importance, e.g. for population dynamics [57], and molt appears to be a potent modulator of such inter-season connectivity.

Our results have implications for (1) the evolution of feather trait diversity among avian taxa and (2) selective forces acting on annual routines. These results may stimulate future inquiry. First, based on current knowledge, we argue that constrained molt could be a more general mechanism at least among small-sized birds that molt their whole plumage once each year. Poor-quality flight and body feathers might have a marked imprint on the residual fitness, which provides a substrate for evolution [58] given the inheritance of feather traits, molt timing and the feather growth rate [34], [39], [59]–[62]. Consequently, species that thrive in different environmental conditions and have diverse annual routines may be exposed to species-specific selective forces, which act on molt rate- and condition-mediated feather quality, and this could have led to the perplexingly high diversity in feather shape, structure and their material properties (e.g. [63]). Second, the mismatch between the timing of parental labor and food peaks due to climate change could drive population declines [64], [65]. Besides this climate-driven selection, our results suggest that there may be an additional selective advantage of early breeding to acquire better quality feathers via a slower molting rate. Sedentary birds may benefit in terms of molting due to the progressive advancement of spring. By contrast, this may have a highly negative impact on summer-molting migrants due to the mismatch of the timing of breeding and food abundance, which might be followed by compromised condition and molting. These aspects suggest that pluralistic approaches are needed by also looking through the prism of molting to better understand eco-evolutionary processes.
